# Genetic and Epigenetic Biomarkers for the Early Oral Cancerization Risk in Periodontitis Patients

**DOI:** 10.3390/cimb47110933

**Published:** 2025-11-09

**Authors:** Giorgia M. Marmo, Morena Munzone, Alessandro Polizzi, Roberto Campagna, Marco Mascitti, Gaetano Isola

**Affiliations:** 1 Department of General Surgery and Surgical-Medical Specialties, School of Dentistry, University of Catania, 95100 Catania, Italy; giorgiamarmo284@gmail.com (G.M.M.); morenamunzone@gmail.com (M.M.); gaetano.isola@unict.it (G.I.); 2 International Research Center on Oral and Periodontal Health “PerioHealth”, University of Catania, 95100 Catania, Italy; 3 Department of Clinical Specialistic and Dental Sciences, Polytechnic University of Marche, 60100 Ancona, Italy; r.campagna@univpm.it (R.C.); marcomascitti86@hotmail.it (M.M.)

**Keywords:** oral cancer, periodontitis, biomarkers, salivaomics, proteomics, oral squamous cell carcinoma, chronic inflammation, oral dysbiosis

## Abstract

Oral squamous cell carcinoma (OSCC) remains one of the most prevalent and aggressive malignancies worldwide, with late diagnosis contributing to poor survival rates. Recent evidence suggests that periodontitis may act as a co-factor in development of OSCC through persistent inflammation, microbial dysbiosis, and subsequent tissue remodeling. Identifying molecular signatures that link periodontitis with early oral cancerization is therefore of paramount importance for risk assessment, prevention, and timely intervention. This narrative review aims to provide an integrative overview of the current knowledge on molecular, genetic, and epigenetic biomarkers associated with oral cancer risk in patients with periodontitis. Specifically, periodontal pathogens such as *Porphyromonas gingivalis* and *Fusobacterium nucleatum* promote oral cancerization by modulating molecular, genetic, and epigenetic pathways, including p53, Cyclin D1, Ki-67, p16^INK4A^, DNA methylation, histone modifications, and microRNA regulation. Therefore, this review provides a discussion about the role of inflammatory mediators, oxidative stress-related molecules, microbial-derived products, genetic markers and epigenetic mechanisms as early molecular signals of malignant transformation. The study of these salivary biomarkers (salivaomics) has emerged as a promising non-invasive diagnostic tool, although variability in sampling, biomarker stability, and confounding factors such as coexisting periodontal disease remain significant limitations. By synthesizing the available evidence, this review summarizes recent evidence linking periodontitis to oral cancerization, highlights potential salivary, proteomic, and inflammatory biomarkers, and considers the role of periodontal therapy in improving inflammatory profiles and modulating tumor-related biomarkers. Finally, it explores future perspectives, including the integration of Artificial Intelligence (AI) to enhance biomarker-based diagnosis and risk stratification in OSCC patients.

## 1. Introduction

Periodontitis is a chronic, multifactorial inflammatory disease mediated by the host immune response to bacterial colonization of the periodontium and related dysbiosis, which, if not preventively treated, can lead to periodontal tissue destruction and tooth loss [1]. Periodontitis is the 6th most prevalent disease worldwide, with moderate forms affecting approximately 50% of the global population [2]. However, persistent inflammation, coupled with the release of tissue degradation products and nutrients, fosters the overgrowth of highly pathogenic bacterial species and leads to clinical signs of periodontal attachment loss [3]. During periodontitis, the bacteria present in the periodontal pocket can also spread out in the bloodstream, resulting in bacteremia and the onset or exacerbation of preexisting inflammatory conditions [2]. This, together with the establishment of a systemic pro-inflammatory state, may underlie the association between periodontitis and various systemic diseases—a topic extensively debated in the literature, particularly over the last few decades [2,4]. Among principal systemic implications are cardiovascular, cerebrovascular, neurological, metabolic, and oncologic disorders [5,6,7,8,9]. Although the precise mechanisms driving carcinogenesis remain incompletely understood, numerous studies have supported a link between periodontitis and several cancer types, including head and neck cancers as well as colorectal, breast, lung and prostate cancers [6,10,11].

Among head and neck squamous cell carcinoma (HNSCC), oral squamous cell carcinoma (OSCC) accounts for approximately 90% of head and neck malignancies and is the sixth most common cancer worldwide, associated with high morbidity, mortality, and recurrence rates [12]. Since 1990, the incidence and the prevalence of oral and lip cancer have increased significantly (by +161.8% and +142.18%, respectively), and projections for 2050 reflect a further rise, with a substantial global impact [13].

Oral hygiene and the composition of the oral microbiota also play a critical role in the risk of developing OSCC. Indeed, patients with OSCC have been shown to exhibit poorer oral hygiene and more advanced periodontitis compared to healthy individuals [14]. Ecological changes affecting the oral microbiome during periodontitis are responsible for the onset of a localized inflammatory state in the head and neck region, particularly within the oral cavity. Nevertheless, the potential for a reverse causal relationship between the two conditions must be considered. Oral squamous cell carcinoma (OSCC), through the induction of pain during toothbrushing, may adversely affect oral hygiene practices. This impairment may facilitate biofilm accumulation, initiate an inflammatory response, and ultimately promote the onset of periodontitis [15].

Although numerous studies have demonstrated an association between periodontitis and oral cancer, it is important to note that such evidence does not establish a definitive causal relationship between the two conditions. One of the main reasons why a clear causal link between periodontitis and oral cancer cannot be asserted is the substantial difference in their respective prevalence rates [16].

The pathogenesis of cancer, as well as the malignant transformation of oral potentially malignant disorders (OPMDs), is strongly associated with the persistence of a chronic inflammatory state, as first demonstrated by Virchow in 1863 through the identification of a lymphoreticular infiltrate within neoplastic tissues [17,18,19,20]. Indeed, the literature supports that prolonged inflammation accounts for approximately 15–20% of malignant tumors [21]. Inflammatory cells and cytokines contribute to the establishment of a favorable tumor microenvironment (TME), which plays a crucial role in cell survival, growth, and proliferation, as well as in the onset of genetic and epigenetic alterations [20]. The mechanisms by which periodontitis and oral bacteria contribute to OSCC development are diverse and include the disruption of epithelial barrier integrity through the infiltration of periodontal pathogens, the induction of persistent inflammation, modulation of signaling pathways, promotion of invasion and metastasis, and alteration of host immune responses [22,23,24]. In this context, periodontopathogenic bacteria exert their effects through a dual mechanism: indirectly, by inducing and perpetuating the immune response; and directly, through specific virulence factors and bacterial metabolic products. The recognition of pathogen-associated molecular patterns (PAMPs) by Toll-like receptors (TLRs) triggers the release of a cascade of pro-inflammatory cytokines (TNF-α, IL-1β, IL-6, and IL-8) and the recruitment of neutrophils, leading to the initiation of the immune response, followed by complement activation and adaptive immunity [25,26]. The chronicity of inflammation is sustained by the persistence of risk factors, such as dysbiosis and immune dysregulation, which lead to cellular proliferation, inhibition of apoptosis, and DNA damage—features commonly implicated in the pathogenesis of various non-communicable diseases, including OSCC [22,27].

Moreover, individual periodontal pathogens are characterized by distinct pathogenic factors that modulate apoptotic pathways, DNA repair mechanisms, and immune evasion. The most prevalent microorganisms in OSCC, *Porphyromonas gingivalis* and *Fusobacterium nucleatum*, exhibit high pathogenic potential and contribute to tumorigenesis through molecular mechanisms involved in cancer initiation, progression, and tumor dissemination [12]. Recent studies have shown increasing interest in tissue-resident microorganisms and their role in modulating inflammatory cytokines and factors involved in immune evasion [24].

Indeed, *P. gingivalis* and *F. nucleatum* exhibit multiple virulence determinants, including lipopolysaccharide (LPS) for immune recognition, toxins (gingipains) mediating tissue damage, and fimbriae and adhesins (FadA) facilitating adhesion and colonization [6,17,28,29]. Oral dysbiosis thus represents a substantial cofactor in cancer development, acting synergistically with other well-established risk factors such as tobacco and alcohol consumption [26,30,31]. Furthermore, periodontal pathogens induce significant alterations in molecular and epigenetic biomarkers implicated in carcinogenesis [29].

The diagnosis of OSCC is often delayed and this condition is mostly detected at its final stages (III or IV), which decreases the survival rate and leads to a noticeable decline in quality of life [32,33,34]. According to Carreras-Torres et al., the 5-year survival rate for OSCC has remained around 50% over the past decades [35]. A systematic review by Grafton Clarke et al. reported that survival is markedly higher in early-stage disease (>80%) but falls below 30% in advanced cases. Diagnostic delay is often attributable to two levels: the patient level and the healthcare professional level [36].

Although tissue biopsy remains the gold standard for diagnosing OSCC, there has been a significant transition from histopathological to molecular diagnostic approaches [37]. This shift is driven both by enhanced understanding of the molecular mechanisms underlying carcinogenesis and by the advent of advanced analytical technologies [38]. In recent years, emerging biomarkers have been identified for the stratification of malignant transformation risk, including alterations in the oral bacteriome and other molecular signatures within the TME [30]. Identifying OSCC biomarkers can help prevent oral cancer [37]. In fact, they play a central role in early diagnosis, which is often key to effectively treating the disease and intervening at a stage when it is more responsive to treatment [39].

Despite advances in the study and identification of novel salivary biomarkers for non-invasive OSCC diagnosis, their clinical application still faces considerable limitations. Moreover, only a few studies have investigated biomarkers common to both periodontitis and cancer. The main challenges of salivaomics include variability in salivary composition, the sensitivity and specificity of individual biomarkers, technical requirements and high costs, as well as the presence of confounding factors [40,41,42]. Importantly, periodontitis itself is regarded as a confounding factor in OSCC biomarker research, since the levels of certain markers may be affected by its presence [43]. This underscores the urgency of conducting broader studies that account for concomitant inflammatory conditions and periodontal status.

In this context, the present review aims to illustrate and discuss recent evidence regarding molecular, genetic, and epigenetic biomarkers as early risk factors for oral carcinogenesis in patients with periodontitis.

## 2. Periodontal Inflammation and Risk Biomarkers for Oral Cancerization

Assessing the risk of oral cancerization in periodontitis patients typically involves the use of molecular and genetic biomarkers, epigenetic modifications, and emerging novel biomarkers [44,45]. Table 1 summarizes changes induced by periodontal bacteria on the main targets.

**Table 1:** Changes induced by periodontal bacteria.

Periodontal Bacteria	Target	Activity Changes	Effects on Tumorigenesis
*P. gingivalis*	P53	Reduced ↓ [46,47]	Increased proliferation of malignant cells, cellular senescence and DNA damage [48]
*P. gingivalis*/*F. nucleatum*	Cyclin D1	Increased ↑ [47,49,50]	Increased cell proliferation [51]
Periodontal bacteria	Ki-67	Increased ↑ [28,52,53]	Increased cell proliferation [54]
*F. nucleatum*	p16^INK4A^	Increased ↑ [55]	Increased cell proliferation and cellular senescence [56,57]
*F. nucleatum*/*P. gingivalis*	Histone H2A phosphorylation	Increased ↑ [6,58]	Increased cell proliferation [58]
*P. gingivalis*	Promoter hypermethylation	Increased ↑ [47]	Increased cell proliferation [59]
*P. gingivalis*	DNA hypomethylation	Increased ↑ [47,60]	Increased cell proliferation [47,60]
*P. gingivalis*	miR-125a	Reduced ↓ [61]	Increased cell proliferation [61]
*P. gingivalis*	miR-200a	Reduced ↓ [61]	Increased cell proliferation [61]
*P. gingivalis*	miR-21	Increased ↑ [47]	Increased cell proliferation
Periodontal bacteria	hsa-miR-224	Increased ↑ [62]	Increased cell proliferation [62]
Periodontal bacteria	hsa-miR-210	Increased ↑ [62]	Increased cell proliferation [62]
Periodontal bacteria	hsa-miR-31	Increased ↑ [62]	Increased cell proliferation [62]
Periodontal bacteria	hsa-miR-497	Reduced ↓ [62]	Increased cell proliferation [62]
Periodontal bacteria	hsa-miR-29c	Reduced ↓ [62]	Increased cell proliferation [62]
Periodontal bacteria	hsa-miR-486	Reduced ↓ [62]	Increased cell proliferation [62]
Periodontal bacteria	miR-19b-3b	Increased ↑ [58]	Increased cell proliferation [26]
Periodontal bacteria	miR-181b-2-3p	Controversial ↑ [63]	Increased cell proliferation [63]
Periodontal bacteria	miR-495-3p	Controversial ↑ [64]	Increased cell proliferation [64]
*P. gingivalis*	MMP-1	Increased ↑ [65]	Extracellular matrix degradation, tumor cell invasion, and metastasis [65]
*P. gingivalis*	MMP-3	Increased ↑ [65]	Increased cell proliferation, tumor cell invasion and metastasis [65]
Periodontal bacteria	MMP-8	Increased ↑ [65]	Controversial role [65]
Periodontal bacteria	MMP-12	Increased ↑ [58]	Increased cell proliferation, tumor cell invasion [65]
*F. nucleatum*	MMP-13	Increased ↑ [58]	Increased cell proliferation, tumor cell invasion and metastasis [65]
Periodontal bacteria	NETs	Increased ↑ [58]	Extracellular matrix degradation, tumor cell adhesion, invasion, and metastasis [66]
Periodontal bacteria	IL-1β and IL-8	Increased ↑ [58]	Increased cell proliferation and angiogenesis [19,67]
*P. gingivalis*	IL-6	Increased ↑ [19,67]	Increased cell proliferation and angiogenesis [19,67]

### 2.1. Molecular Biomarkers

Periodontitis and oral preneoplastic and neoplastic disorders can share several molecular biomarkers, including proteins and mediators implicated in the proliferation of cancer cells. Alterations in the levels of these biomarkers have been observed in patients with periodontitis and OSCC [46,47].

**Figure 1:**
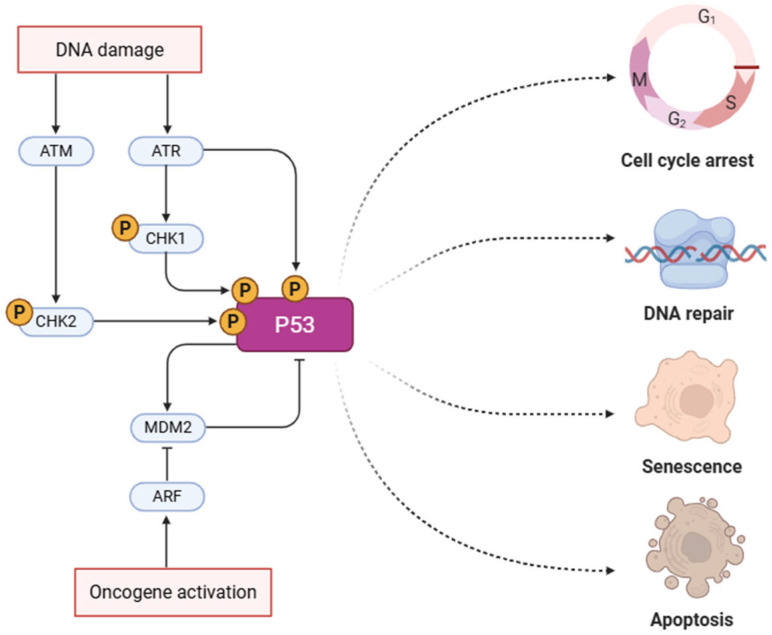
P53 pathway in oncogenesis. This pathway highlights the activation of p53 in response to DNA damage or oncogene activation. DNA damage activates ATM/ATR kinases, which phosphorylate CHK1/CHK2 and subsequently p53. Stabilized p53 directs the cell toward one of several outcomes: cell cycle arrest, DNA repair, senescence, or apoptosis, depending on the context and damage severity [74]. Created in https://BioRender.com, accessed on 24 May 2025.

The p53 protein is a transcription factor encoded by the tumor-suppressor gene *TP53*, which plays a crucial role in genome defense [12]. It involves aging phenomena and senescence, cellular differentiation, cell cycle regulation, apoptosis, and DNA repair (Figure 1). Mutations in the encoding gene (due to deletion or demethylation) are implicated in the development of various malignancies, including OSCC [29,46]. Chattopadhyay et al. reported the presence of *TP53* mutations in exon 4, at codon 63, in the saliva of patients with OSCC, as detected through circulating tumor DNA (ctDNA) analysis [59]. Mutations affecting *TP53* have been linked to OSCC, particularly in the early stages of tumor development [68]. Specifically, a study by Jagadeesan et al. found that approximately 80% of oral carcinomas exhibit a *TP53* mutation, and 10% of dysplasia cases too [12]. According to Radaic et al., *TP53* mutation represents one of the most frequent alterations in oral carcinoma, with a frequency of 75–85% in HPV-negative HNSCC cases [44,69]. Furthermore, the study by Starska-Kowarska, comparing salivary samples from healthy individuals and OSCC patients, confirmed the high prevalence of *TP53* mutations (93.3%) in cancer cases compared to controls [68]. P53 regulates the expression of p16^INK4a^, CDKN2A (cyclin-dependent kinase inhibitor 2A), and p52, and its inactivation prevents cell cycle arrest in the G1 phase, thereby allowing the proliferation of potentially malignant cells. [48]. Additionally, it may impair the ability of cells to repair damaged DNA, predisposing them to further mutations. Studies show how some periodontal pathogens affect p53 activity, resulting in an increased predisposition to carcinogenesis and the malignant transformation of (OPMDs). For example, *P. gingivalis* modulates cell cycle regulation by reducing both the levels and activity of p53 in gingival epithelial cells, an effect induced by the activation of the PI3K/Akt pathway [46,47] (Table 1). Furthermore, LPS from *P. gingivalis* induces senescence in human gingival fibroblasts (HGFs) by increasing p53 concentration [70,71]. The results suggest a potential discrepancy in the effects exerted by *P. gingivalis* on gingival epithelial cells and fibroblasts. Nevertheless, these observations are consistent with recent evidence indicating that *P. gingivalis* can employ distinct strategies to enhance host cell survival, while simultaneously modulating multiple anti-apoptotic signaling pathways [72]. Previous studies have demonstrated that the ability of *P. gingivalis* to elicit either pro- or anti-apoptotic responses is tightly dependent on the specific host cell type and/or the presence of particular bacterial components, such as cysteine proteinases or LPS [72]. Abnormalities in p53 within fibroblasts have been associated with increased tumor aggressiveness, metastasis, and poor response to therapy [73]. Several studies further suggest that p53 may represent a key factor linking periodontal inflammation to carcinogenesis. Indeed, p53 is involved in the antioxidant response, and its levels are influenced by hypoxia and the presence of reactive oxygen species (ROS) [71]. *F. nucleatum* has also been implicated in regulating cell survival and proliferation mechanisms through the Ku70/p53 pathway [47]. In addition, *F. nucleatum* induces DNA damage and downregulation of the *TP53* gene, thus predisposing cells to proliferation [21].

Cyclin D1 is a cell cycle regulatory protein that facilitates the G1-to-S phase transition by binding to CDK4 and CDK6, implicating it in uncontrolled cell proliferation in various tumors [51]. Elevated levels of Cyclin D1 are also associated with a poor prognosis, making it a potential prognostic factor for OSCC when used in conjunction with other biomarkers. Its overexpression has been associated with OSCC and is induced through signaling pathways mediated by NF-kB, STAT3, and TLR2 [28]. Several studies have reported a link between periodontal pathogens and Cyclin D1 levels. The review by Lafuente Ibáñez Mendoza et al. demonstrated that in murine OSCC models, *P. gingivalis* infection leads to an increase in Cyclin D1 [28]. *P. gingivalis* infection induces inflammatory cytokines (e.g., IL-6), activating NF-kB and STAT3, which in turn upregulate Cyclin D1 [28,75,76].

Similarly, *P. gingivalis* can raise Cyclin D1 levels through a negative feedback mechanism on the miR-21/PDCD4/AP-1 pathway [47] (Table 1). *F. nucleatum* infection has been associated with elevated Cyclin D1 activity in OSCC, accelerating cell cycle progression and contributing to increased cell proliferation [49,50] (Table 1). Moreover, the adhesin FadA of *F. nucleatum* promotes colorectal cancer oncogenesis through the expression of annexin 1 (ANXA1) and Cyclin D1 [77,78].

Ki-67 is a nuclear protein involved in the active phases of the cell cycle and cellular proliferation and is expressed in all proliferating cells [54]. Its overexpression has been associated with inflammatory states and increased invasiveness in various cancers, including but not limited to OSCC (Table 1). Several studies have recognized its role as both a diagnostic and prognostic biomarker. Under physiological conditions, Ki-67-positive cells are confined to the basal layer of the epithelium; however, in potentially malignant and malignant lesions, its expression extends above the basal layer and is significantly increased [76]. Furthermore, Ki-67 may serve as a predictive marker for treatment response. When assessed in combination with p16^INK4A^, it has proven to be a more reliable and valid biomarker compared to p16 alone. In particular, OSCC tumors characterized by low Ki-67 expression and high p16 levels (p16+/Mib−) demonstrated superior overall survival rates (83% at 5 years) and improved disease-free survival compared to other subgroups, such as p16+/Mib+ tumors (25% at 5 years) [49]. However, according to Jagadeesan et al., when evaluated in isolation, Ki-67 exhibits a weaker correlation with OSCC compared to other biomarkers [12]. Salivary Ki-67 has been detected at high concentrations in inflammatory conditions and has been positively correlated with the severity of periodontitis [76]. The association between periodontal inflammation and Ki-67 levels may be explained by the presence of inflammatory mediators and signaling factors that contribute to the establishment of a pro-tumorigenic microenvironment in response to periodontal pathogens. These factors—including platelet-derived growth factor (PDGF), nitric oxide (NO), transforming growth factor-beta (TGF-β), hepatocyte growth factor (HGF), and nuclear factor kappa-light-chain-enhancer of activated B cells (NF-κB)—can induce Ki-67 overexpression, thereby predisposing tissues to the onset and progression of OMPDs and OSCC [76]. Periodontal pathogens can also modulate Ki-67 levels, thereby promoting tumorigenesis. Indeed, studies have shown that in murine models, *T. denticola* enhances tumor progression via the TGF-β signaling pathway and increased Ki-67 expression [52]. It has also been hypothesized that the upregulation of Ki-67 may be closely linked to *P. gingivalis*-induced NF-kB activation in oral epithelial cells exposed to periodontal pathogens [28] (Table 1). Another study on murine models of periodontitis demonstrated a correlation between elevated Ki-67 proliferation index and OSCC development, with significantly higher levels in advanced stages, associated with increased tumor burden, enhanced cellular proliferation, and decreased survival rates [53] (Table 1).

**Figure 2:**
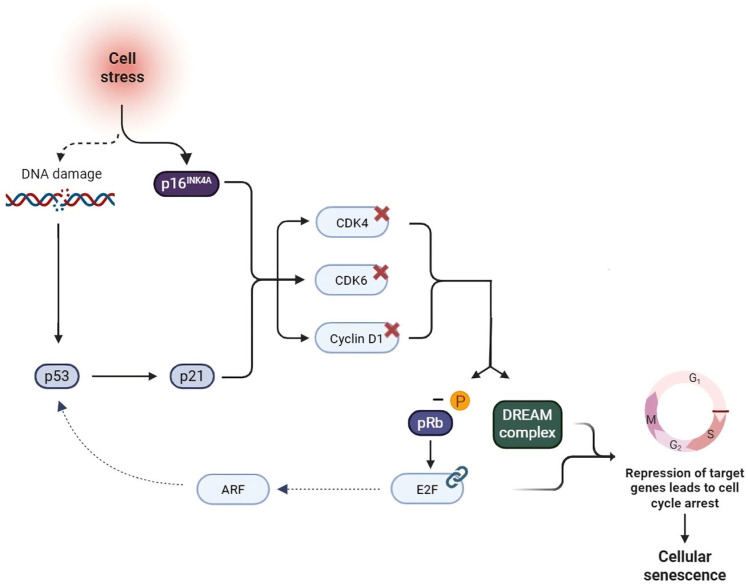
p16^INK4A^/pRB and p53/p21 pathways in cellular senescence. Cellular stress, genomic damage, telomere shortening, and inflammatory cytokines contribute to the activation of the p16^INK4A^/pRB and p53/p21 pathways, and the DREAM complex (a multiprotein complex composed of DP, Rb-like, E2F, and MuvB) [56]. The upregulation of p16 leads to the inhibition of CDK4–6 and Cyclin D1, resulting in the dephosphorylation of pRB. In its active form, pRB binds to the transcription factor E2F, which, together with the DREAM complex, represses the transcription of target genes leading to cell cycle arrest [56]. Concurrently, p53 promotes the expression of dephosphorylated pRB through the activation of p21, further contributing to the establishment of cellular senescence. Moreover, a functional link between the two pathways has been identified, mediated by the transcription factor E2F, which activates the tumor suppressor gene *ARF*, thereby inhibiting MDM2 and stabilizing p53 [57].Created in https://BioRender.com, accessed on 29 October 2025.

The p16^INK4A^ protein is a key cell cycle regulator that inhibits the transition from G1 to S phase by suppressing cyclin-dependent kinases CDK4 and CDK6 [76]. Its overexpression is associated with uncontrolled cellular proliferation and the development of multiple cancers, including OSCC. P16 is one of the principal biomarkers of cellular senescence (Figure 2). Cellular senescence represents a manifestation of cellular aging and is characterized by metabolic and structural alterations, resistance to apoptosis, and permanent cell cycle arrest. The persistence of senescent cells may influence neighboring cells by promoting chronic inflammatory processes [1]. Moreover, p16-rich cells have been implicated in the development of various chronic diseases. Recent evidence has demonstrated that the periodontal microenvironment can induce premature senescence through inflammation and oxidative stress, thereby predisposing individuals to the onset of disease even at a young age [1]. Exposure to periodontal pathogens—such as *Fusobacterium nucleatum*—has been shown to induce senescence-like features in gingival keratinocytes, accompanied by increased p16 expression. Epithelial cell senescence reduces their intrinsic repair capacity, thereby compromising periodontal homeostasis and increasing susceptibility to tissue damage [55]. In cases of chronic and persistent injury, these cells tend to accumulate and exert pro-tumorigenic effects due to the acquisition of the so-called senescence-associated secretory phenotype (SASP), which is characterized by altered gene expression and the release of inflammatory mediators. The SASP, within the context of carcinogenesis, promotes the development of epithelial tumors including OSCC [79]. By regulating proteins known as cyclin-dependent kinase inhibitors, including p16^INK4a^ and p14^ARF^, senescence leads to an irreversible arrest of the cell cycle. According to Albuquerque-Souza et al., also *F. Nucleatum* induces cellular senescence through the upregulation of p16^INK4a^. This mechanism highlights the carcinogenic potential of this microorganism. Furthermore, it plays a fundamental role in HPV-related cervical and oropharyngeal cancers, in conjunction with the underexpression of retinoblastoma protein (pRb) [55] (Table 1). Immunohistochemical analyses of OSCC have demonstrated an association between P16 and P53 expression with more aggressive lesions, lymphovascular invasion, and poor prognosis [73]. In HPV infection cases, P16 and pRb levels may serve as prognostic factors; however, conclusive studies on this subject are still lacking [48,80]. The role of p16 as a biomarker in OSCC remains controversial due to inconsistent findings. Loss of p16 expression has been reported in early stages of OSCC development, whereas its positivity does not appear to correlate reliably with histopathological diagnosis [76]. Although p16 alone has demonstrated minimal prognostic relevance in OSCC, a study by Richter et al. suggests that, when used in combination with Ki-67, it may serve as a potential predictor of survival and malignant transformation [54].

### 2.2. Epigenetic Changes and Genetic Biomarkers

Epigenetics refers to the study of reversible and heritable changes in gene expression not encoded within the DNA sequence. Epigenetic mechanisms include DNA methylation, histone modifications, and microRNAs [81,82]. Such modifications influence gene expression by inducing activation or silencing, with DNA methylation and histone modifications playing key roles in oncogenesis and potentially representing a point of convergence between periodontitis and OSCC development [51]. Indeed, oral bacteria such as *P. gingivalis* and *F. nucleatum* have been shown to trigger epigenetic alterations in gingival epithelial cells [81]. For example, both species have been associated with up to 125-fold increases in OSCC cell proliferation through histone H2A phosphorylation, leading to the downregulation of p53 and Ku70 pathways [6] (Table 1).

Promoter hypermethylation, which induces gene silencing, represents an early event in OSCC development. The inactivation of tumor suppressor genes such as *CDKN2A*, *hMLH1*, and *hMSH2* promotes tumor progression [51]. Identifying hypermethylated genes involved in proliferation, adhesion, DNA repair, and angiogenesis may provide a valuable tool for early OSCC diagnosis [59]. Several studies have reported frequent hypermethylation events in the 9p21 chromosomal region, encompassing the p14ARF, p15INK4b, and p16^INK4a^ gene clusters [68]. Loss of *CDKN2A* function and its encoded protein, p16, has been observed in 80% of OSCC cases [83]. Conversely, hypomethylation can activate oncogenes, increasing cancer risk [51]. For example, *P. gingivalis,* through its virulence factors (particularly LPS), induces an increase in IL-6 that drives epigenetic changes—including promoter hypermethylation and DNA hypomethylation—thereby facilitating cellular replication [47,60] (Table 1). A bioinformatics analysis by Li et al. revealed overlapping methylation patterns between periodontitis and OSCC. Specifically, altered methylation profiles (not limited to hypermethylation) were identified in genes such as *MPPED1*, *PROC*, *TUBA4B*, *PLD6*, *RSPH4A*, *RSPH9*, and *CSPG4* in both conditions [84]. Similarly, a review by Lavu et al. reported comparable proportions of hypermethylation in E-cadherin and COX-2 in patients with periodontitis and breast cancer, suggesting a potential correlation [81].

Histone modifications, including acetylation and methylation, influence chromatin remodeling and transcription regulation. Acetylation facilitates transcription, whereas deacetylation suppresses it. Other modifications, such as methylation, phosphorylation, and ubiquitination, differentially affect chromatin architecture and gene expression [51]. *F. nucleatum* and *P. gingivalis* have been shown to downregulate the p53 and Ku70 signaling pathways through histone H2A phosphorylation, thereby promoting increased cell proliferation in OSCC cells [58] (Table 1).

Ribosomal microRNAs (miRNAs) found in saliva are small non-coding RNAs (20–22 nucleotides) involved in gene regulation. These molecules influence cell differentiation, proliferation, apoptosis, and migration, with expression patterns akin to oncogenes and tumor suppressors [12]. Based on their activity, miRNAs are classified as oncogenic (also known as oncomirs), tumor suppressors, prometastatic (metastamiRs), and metastasis suppressors [58].

Notably, miR-125a and miR-200a, which are downregulated in periodontitis as a result of *P. gingivalis* exposure, are also found to be downregulated in OSCC patients, suggesting a potential tumor-suppressive role [61] (Table 1 and Table 2). In addition, another study reports the high accuracy of miRNA-136, miRNA-27B, and miR-27b in saliva for the diagnosis of OSCC [85].

Salivary rinse or oral brushing can detect methylated genes linked to OSCC with high sensitivity (≥75%) and specificity (≥90%) [47]. *P. gingivalis* can indeed promote the proliferation of tumor cells in OSCC through the modulation of miRNA expression, particularly increasing miR-21, which deregulates the miR-21/PDCD4/AP-1 pathway [47] (Table 1).

A study by Chen et al. demonstrated a significant association among periodontitis, OSCC, and three miRNAs: hsa-miR-19b-3p, hsa-miR-181b-2-3p, and hsa-miR-495-3p. Hsa-miR-19b-3p is associated with overall survival in oncology patients and is found in high concentrations in those with periodontitis [26]. Similarly, hsa-miR-181b-2-3p, an oncogenic miRNA, is upregulated in OSCC patients. miR-181b-2-3p has been identified as a reliable marker for lymph node metastasis, together with three other miRNAs (miR-21-5p, miR-107, miR-1247-3p) [63]. However, there are no definitive studies yet regarding changes in miR-181b expression levels in relation to disease progression. In fact, miR-181b appears to increase progressively with the severity of epithelial dysplasia, subsequently decreasing in OSCC samples. Therefore, miR-181b may act during the early stages of epithelial dysplasia development and later undergo suppression following malignant transformation. Conversely, in leukoplakia samples with low-grade dysplasia, miR-181b appears to be downregulated. Overall, current evidence highlights a substantial variability in the mechanisms of action of the different miR-181b isoforms, depending on the tumor microenvironment context. Further studies are needed to better elucidate their precise role in oral carcinogenesis [86]. Although hsa-miR-495-3p exhibits anti-oncogenic activity in various cancers, in the case of OSCC it appears to serve as a reliable prognostic risk factor, with elevated levels indicative of a poorer prognosis [26] (Table 1 and Table 2). The role of mir-495 remains rather controversial, as the available evidence presents highly conflicting data regarding its expression in OSCC samples. According to a study by Lv et al., miR-495 is downregulated in OSCC and, in vitro, limits OSCC cell proliferation and invasion through the repression of its target Notch1. Similarly, in gastric cancer, it appears to exert a tumor-suppressive effect [64]. The findings of You et al. support these results, demonstrating that miR-495 inhibits EMT, proliferation, migration, and invasion of cancer stem cells in OSCC by regulating the TGF-β signaling pathway [87].

A network analysis by Li et al. identified 18 co-expressed miRNAs in both OSCC and periodontitis, implicated in immune, inflammatory, and carcinogenic responses. Among them, three were co-upregulated (hsa-miR-224, hsa-miR-210, hsa-miR-31) and three co-downregulated (hsa-miR-497, hsa-miR-29c, hsa-miR-486). Notably, hsa-miR-224 and hsa-miR-31 emerged as reliable biomarkers for OSCC [62] (Table 2).

Periodontal pathogens and their carcinogenic metabolites are responsible for the genetic and epigenetic alterations underlying tumorigenesis [84]. Oxidative stress, a consequence of chronic periodontal inflammation, promotes the release of ROS, such as superoxide radicals and hydrogen peroxide, as well as reactive nitrogen intermediates (RNI), including nitric oxide and peroxynitrite [22,23,88]. These free radicals modify and damage DNA, thereby predisposing to tumorigenesis [22]. DNA damage—including base-pair mismatches, replication errors, oxidative deamination, and hydrolysis—can impair normal mechanisms of cell cycle regulation, DNA repair, and apoptosis, and may result from oxidative stress [89]. The most frequent mutation detected in OSCC samples involves the *TP53* gene (93.3%). Mutation of the p53 protein represents an early biomarker of oral cancer [68].

Mutations in tumor suppressor genes and chromosomal abnormalities contribute to increased cancer susceptibility [83]. Identifying these genetic alterations aids in patient risk stratification [90]. These genetic aberrations include mutations, loss of heterozygosity (LOH) and alterations in DNA repair mechanisms [45].

The relationship between tumor suppressor gene (TSG) mutations and the levels of genetic and epigenetic biomarkers has not yet been fully elucidated in the literature. Mutations in TSGs directly affect cellular proliferative activity and, consequently, the levels of proliferative biomarkers [91]. For instance, mutation of the *CDKN2A* gene directly impacts the p16^INK4A^/pRB pathway through the activation of the transcription factor E2F, resulting in increased cellular proliferation [56]. Furthermore, an inverse causal relationship also exists between epigenetic alterations and TSG mutations. Indeed, aberrant hypermethylation and consequent silencing of genes involved in DNA repair mechanisms lead to high genomic instability, which in turn contributes to the accumulation of additional mutations and epigenetic modifications [92].

LOH refers to the loss of an allele at a chromosomal locus, resulting in gene function loss [74]. A high incidence of loss of heterozygosity (LOH) has also been observed in salivary and tissue samples of OSCC patients at chromosomal regions 9p, 3p, and 17p [68].

In OSCC, LOH frequently affects the *CDKN2A* gene on chromosome 9p, encoding the p16 protein [56]. LOH in OSCC also impacts chromosome 3p, which harbors genes involved in cellular proliferation, DNA synthesis, and cellular adhesion. Chromosome 3p alterations are associated with survival and recurrence risk [83]. This condition can arise from various mechanisms, including coding region mutations, promoter methylation, chromosomal rearrangements, and DNA repair defects [83]. Interestingly, LOH tends to occur more frequently at specific loci, associated with risk factors such as tobacco and alcohol consumption [68]. Future research may investigate the role of periodontal pathogens as potential risk factors for LOH.

### 2.3. Emerging Biomarkers

Recent research has explored novel biomarkers, including salivary and proteomic epigenetic mediators, which offer potential for high-sensitivity and high-specificity biomarker for disease detection [12,93].

Liquid biopsy, which involves the analysis of biological fluids, and salivaomics, the study of salivary omics markers, represent innovative, non-invasive techniques for the early detection of neoplasms. Salivaomics encompasses multiple domains, including genomics, transcriptomics, proteomics, epigenomics, metabolomics, metagenomics, and microbiomics. Saliva collection and analysis represent a promising and cost-effective strategy for OSCC diagnosis [68,94].

Proteins secreted by TME cells provide valuable insights into tumor progression [95]. Saliva contains a diverse range of proteins that have been extensively studied as potential biomarkers [44]. TME proteomics has identified proteins involved in metabolic pathways (e.g., glucose metabolism), extracellular matrix remodeling, and hypoxia [51]. These biomarkers can be employed not only for assessing disease progression but also for evaluating associated risk factors [44].

Notably, elevated concentrations of metalloproteinases (MMPs) and neutrophil elastase, characteristic of periodontitis, have been linked to OSCC and its recurrence [96].

MMPs are implicated in carcinogenesis, angiogenesis, and metastasis. Chen et al.’s study revealed elevated levels of MMP-12 and MMP-13 in OSCC patients, demonstrating that these enzymes may serve as diagnostic and prognostic biomarkers [26]. Furthermore, the expression of MMP-13 is closely linked to the activation of oncogenic-related pathways (NF-κB or MAPK/p38 pathways) by *F. nucleatum* [58] (Table 1). Periodontal inflammation mediated by neutrophils induces the secretion of elastase, one of the protein components of NETs [26] (Table 1). Several studies have demonstrated the role of neutrophil elastase in extracellular matrix degradation, tumor cell adhesion, invasion, and metastasis in OSCC [66] (Table 2). For instance, a study by Monea et al. demonstrated the upregulation of MMP-1, MMP-2, MMP-3, MMP-8, MMP-9, MMP-10, MMP-12, and MMP-13 in samples from oral cancer patients, with MMP-1, MMP-3, and MMP-9 identified as promising diagnostic biomarkers [65]. Moreover, a study by Jansson et al. demonstrated the upregulation of MMP-1, MMP-2, MMP-3 in gingival crevicular fluid from periodontitis patients [97] (Table 1 and Table 2). Notably, MMP-8, a key mediator of tissue destruction in periodontitis, is a shared biomarker between periodontitis and OSCC [65] (Table 2).

Cytokines and inflammatory mediators also represent promising biomarkers in oral cancer and are closely associated with periodontitis. IL-6 and IL-8, NF-kB-dependent cytokines, are central messengers involved in inflammatory responses, cell proliferation, and angiogenesis, and serve as sensitive early biomarkers for OSCC [19,67].

IL-1β and IL-8, key mediators of periodontal inflammation, are also involved in OSCC. Specifically, IL-1β plays a critical role in both acute and chronic inflammatory responses and promotes cell proliferation. Its levels are elevated in patients with periodontitis and OSCC, suggesting its potential use as a biomarker. IL-8 (CXCL8) is essential for neutrophil and granulocyte chemotaxis and contributes to disease progression and metastasis through the activation of the NF-κB signaling pathway [26] (Table 2).

**Table 2:** Differential expression patterns of biomarkers in both periodontitis and OSCC.

Target	Expression Pattern in Periodontitis	Expression Pattern in OSCC
miR-125a	Reduced ↓	Reduced ↓ [61]
miR-200a	Reduced ↓	Reduced ↓ [61]
miR-19b-3p	Increased ↑	Increased ↑ [26]
miR-181b-2-3p	Reduced ↓	Controversial [26,63]
miR-495-3p	Reduced ↓	Controversial [86]
hsa-miR-224	Increased ↑	Increased ↑ [62]
hsa-miR-210	Increased ↑	Increased ↑ [62]
hsa-miR-31	Increased ↑	Increased ↑ [62]
hsa-miR-497	Reduced ↓	Reduced ↓ [62]
hsa-miR-29c	Reduced ↓	Reduced ↓ [62]
hsa-miR-486	Reduced ↓	Reduced ↓ [62]
MMP-1, MMP-2, MMP-3	Increased ↑ [97]	Increased ↑ [65]
MMP-12	Increased ↑	Increased ↑ [58]
MMP-13	Increased ↑	Increased ↑ [58]
MMP-8	Increased ↑	Increased ↑ [65]
NETs	Increased ↑	Increased ↑ [96]
IL-1β	Increased ↑	Increased ↑ [58]
IL-6	Increased ↑	Increased ↑ [19,67]
IL-8	Increased ↑	Increased ↑ [58]

### 2.4. The Role of Periodontal Therapy in Biomarker Levels

Periodontal therapy significantly reduces both local and systemic inflammation, improving inflammatory markers and modulating tumor-related biomarkers. Furthermore, non-surgical periodontal therapy aims to restore a balanced condition from both an immunological and microbiological perspective; focusing on microbiome-driven immunological pathways represents a promising therapeutic strategy for the management of chronic periodontitis and OSCC [15,98,99].

Mechanical instrumentation and plaque removal lead to a decrease in IL-6 and IL-8 levels, while the oral microbiome benefits from a decrease in periodontal pathogens [100]. While in gingival crevicular fluid (GCF), a reduction in local levels of pro-inflammatory cytokines such as IL-1β, TNF-α, IL-17, and IL-23, as well as other mediators including MMP-8 and VEGF, is observed [101,102]. VEGF plays a crucial role in OSCC, as it is involved in both angiogenesis and tumor progression [46].

Moreover, periodontal treatment contributes to the downregulation of TNF-α, MMP, and pro-inflammatory cytokines and it significantly influences neutrophil ROS production, thereby reducing NETs formation and the consequent oxidative stress and tissue damage [98,100,103].

Recent studies have highlighted the influence of non-surgical periodontal therapy on miRNA levels, particularly miR-21, which regulates key signaling pathways involved in tumor progression [58,85]. Indeed, if the expression of specific miRNAs (e.g., miR-21, miR-146a, and miR-155) is strongly correlated with IL-1β or TNF-α levels in periodontitis, the elimination of such pro-inflammatory stimuli following periodontal therapy could lead to a significant reduction in their expression [104].

*P. gingivalis* modulates the expression of miRNA-205-5p, which is known for its immunomodulatory properties, in gingival epithelial cells. As there is a reduction in bacterial load of *P. gingivalis* after mechanical debridement, the level of miRNA-205-5p expression also tends to normalize, thereby contributing to the restoration of balance in the inflammatory response [29,105].

Current evidence does not provide direct proof of the role of periodontal therapy in the suppression of OSCC. Periodontal therapy represents a crucial tool in the primary prevention of OSCC by targeting oral dysbiosis and both local and systemic low-grade chronic inflammation [28]. At present, oral cancer treatment relies on conventional approaches such as surgical excision, radiotherapy, and chemotherapy, as well as innovative strategies including immunotherapy and molecular targeting [90,106]. Considering the beneficial effects of periodontal management on inflammatory mediators and biomarkers, scaling and root planing may influence disease progression and reduce tumor aggressiveness.

Recent studies have demonstrated that inflammatory mediators play a critical role in the progression of OSCC, indeed persistent exposure to inflammatory signals promote chemoresistance and enhance the aggressiveness of tumor cells. In particular, IL-6 has been implicated in conferring resistance to paclitaxel. These findings suggest that periodontitis-associated pathogens and inflammatory mediators are key contributors to the development of chemoresistance in OSCC [107].

In light of these considerations, an approach that integrates periodontal therapy into the management of patients with periodontitis appears to be a prudent and potentially effective strategy for preventing the onset and progression of OSCC [46,108].

## 3. Current Limitations

The research of biomarkers has a pivotal role for an early diagnosis, which is often the key in effectively treating a disease and it can help to intervene at a stage when the disease is more responsive to treatment [48]. For the study of tumor, its possible to use markers in serum, tissue, and other body fluids. However, the investigation of blood biochemical have some disadvantages, in fact blood sampling is an invasive procedure and has a potential risk of disease transmission through needle stick injuries. For this reason, saliva is used for non-invasive diagnostic medium [109]. The use of salivary samples for biomarker research nonetheless presents certain limitations: the methods of sampling and of nucleic acid extraction and analytical procedures can distort the results too, like stimulated and unstimulated saliva, the time of collection, pH, and flow rate, oral rinse, oral swab, scraping, cytobrush, and biopsy [30,68]. Moreover, the concentration and stability of biomarkers in salivary samples are influenced by several unhealthy lifestyle behaviors, such as alcohol consumption, smoking, as well as by systemic diseases, pharmacological treatments, radiotherapy, the presence of enzymes and the condition of the gums and teeth [68]. Finally, to establish saliva as a reliable medium for clinical diagnostics, it is necessary to standardize the saliva collection method, processing, analysis, and reporting of liquid biopsy results [94].

Among the limitations is the lack of long-term longitudinal studies too. Indeed, in the absence of such studies, it is difficult to definitively establish a strong correlation between periodontitis and cancer, control for confounding factors, and assess the benefits of periodontal therapy [15].

## 4. Future Directions

Since biomarker assessment and the role of periodontal therapy, alongside traditional risk predictors of cancerization, enables optimal patient care and more precise risk stratification, clinically detected factors and biomarkers may be incorporated into artificial intelligence (AI) algorithms to assist clinicians in diagnosis and risk stratification [93,110].

One of specialized algorithms such as machine learning (ML) can identify patterns and relationships within datasets, while deep learning (DL) employs complex multilayer neural networks capable of analyzing intricate data structures. These technologies have various applications: ML can aid in the identification and validation of biomarkers, support decision-making systems based on patient data, and generate risk scores for malignant transformation [93,111]. Conversely, DL is particularly useful in analyzing histopathological images, distinguishing between benign and malignant lesions, and assessing tumor size and depth of invasion [93]. The integration of AI in clinical practice could enhance the identification and management of oral cancer patients by enabling the development of personalized treatment plans based on individual risk factors [44].

Moreover, recent studies have identified biomarkers in saliva, blood, buccal swabs, and other body fluids, along with promising artificial intelligence (AI) strategies, as potential tools for the early detection of oral cancer [111]. Recent studies have also demonstrated that AI can serve as a non-invasive and cost-effective tool to understand the role of the microbiome in OSCC, thereby improving diagnosis, monitoring progression, and treatment. However, the clinical application of AI requires standardized protocols, diverse patient cohorts, and validation through large-scale longitudinal studies [112].

Starting from the use of salivary samples, the presence of dental diseases such as periodontitis and oral cancer could potentially be diagnosed through metabolomics, which focuses on the qualitative and quantitative analysis of endogenous low-molecular-weight metabolites [68]. Therefore, the application of salivary metabolomics to simultaneously assess a range of metabolites could prove useful for identifying biomarkers the diagnosis and monitoring of OSCC [94].

Finally, the identification of bacterial species capable of promoting carcinogenesis may serve as a valuable diagnostic tool OSCC. Metagenomic analyses have revealed significant differences in the oral microbiota between patients with precancerous lesions or oral cancer and healthy patients. Various technologies have been employed to study the oral microbiota, including culture-based methods, microscopy, DNA microarrays, PCR, 16S rRNA gene sequencing, and high-throughput sequencing. Recent studies have associated the presence of specific bacteria—such as *Prevotella*, *Streptococcus*, *Salmonella*, *Fusobacterium nucleatum*, and *Porphyromonas gingivalis*—with OSCC. Therefore, the analysis of shifts in the composition and activity of oral bacterial communities represents a promising biomarker for the progression of neoplastic lesions in the oral epithelium and for the early diagnosis of OSCC [68].

## 5. Conclusions

This review highlights the current evidence on the status update, emphasizing the common pattern and pathways between chronic periodontal inflammation and oral carcinogenesis. It also highlighted the crucial role of certain molecular mediators, as well as genetic and epigenetic biomarker alterations induced by periodontal pathogens. In this regard, potential clinical patient stratification based on *P. gingivalis* and *F. nucleatum* concentrations in gingival tissues could aid in tumor initiation and progression, modulating pathways related to cell proliferation, apoptosis, DNA repair, and immune evasion.

Furthermore, current evidence suggests that salivary biomarkers, particularly those related to proteomics, hold promising opportunities for the early and non-invasive diagnosis of OSCC. In this regard, periodontal therapy, driven by reducing the expression of key periodontal pathogenic bacteria and the related immune response—a pathway that has been shown to be shared between preneoplastic lesions and OSCC, both in controlling local inflammation and modulating tumor-related biomarkers—has been shown to currently represent one of the main strategies to be applied to reduce various environmental cancer risk factors in patients with periodontitis. Looking ahead, integrating biomarker assessment with artificial intelligence-based diagnostic tools could support and enhance more accurate risk stratification and personalized patient management. Overall, enhancing our understanding of the shared mechanisms between periodontitis and OSCC will be crucial for developing preventive strategies, improving early diagnosis, and ultimately improving patient survival outcomes.

However, potentially effective mechanisms in vitro or on limited samples still need to be better evaluated in larger study populations, as saliva composition has been shown to vary significantly from one sample to another. Therefore, the current methodologies developed in recent years will require further studies to validate their large-scale standardization and better validate their clinical applicability.

## Data Availability

The original contributions presented in this study are included in the article. Further inquiries can be directed to the corresponding author.

## Abbreviations

The following abbreviations are used in this manuscript:

OSCC	Oral squamous cell carcinoma
OPMDs	Oral potentially malignant disorders
HNSCC	Head and neck squamous cell carcinoma
TME	Tumor microenvironment
PAMPs	Pathogen-associated molecular patterns
TLRs	Toll-like receptors
CtDNA	Circulating tumor DNA
HGFs	Human gingival fibroblasts
ROS	Reactive oxygen species
PDGF	Platelet-derived growth factor
NO	Nitric oxide
TGF-β	Transforming growth factor-beta
HGF	Hepatocyte growth factor
NF-κB	Nuclear factor kappa-light-chain-enhancer of activated B cells
pRb	Retinoblastoma protein
MiRNA	microRNA
TSG	Tumor suppressor gene
LOH	Loss of heterozygosity
MMP	Metalloproteinase
NETs	Neutrophil extracellular traps
AI	Artificial intelligence
ML	Machine learning
DL	Deep learning
